# Experience with concurrent chemoradiotherapy treatment in advanced cervical cancer: results from a hospital in Argentina

**DOI:** 10.3332/ecancer.2019.919

**Published:** 2019-04-02

**Authors:** María Eugenia Giavedoni, Lucas Staringer, Rosa Garrido, Cintia Bertoncini, Mabel Sardi, Myriam Perrotta

**Affiliations:** 1 Department of Gynaecology, Hospital Italiano of Buenos Aires, Buenos Aires C1199 ABH, Argentina; 2 Department of Radiation Oncology, Hospital Italiano of Buenos Aires, Buenos Aires C1199 ABH, Argentina

**Keywords:** carcinoma of the cervix, radiotherapy, concurrent chemotherapy, brachytherapy

## Abstract

**Objective:**

To describe our experience with concurrent chemoradiotherapy using three-dimensional conformal radiotherapy (3D-CRT) and high-dose-rate intracavitary brachytherapy with weekly cisplatin in the treatment of patients with locally advanced cervical cancer.

**Methods:**

Forty-three patients were identified between January 2009 and December 2015. Their medical records were retrospectively reviewed, and data on patient characteristics, tumour, treatment and toxicities were collected and analysed.

**Results:**

The median age was 45 years (interquartile range (IQR): 26) The median tumour size was 45 mm (IQR: 20). Thirty-eight patients (88%) had a cervical tumour with a size of ≥ 40 mm. The median cervical tumour size evaluated by magnetic resonance imaging (MRI) was 52 mm (IQR: 17). Twenty-two patients (51%) had enlarged lymph nodes on MRI (≥ 10 mm). MRI demonstrated the involvement of the parametrium in 29 patients (67%). Fifteen patients had positive para-aortic nodes (36%). The median total treatment time was 58 days (IQR: 20). Sixteen patients (39%) received extended-field radiotherapy. Cisplatin was administered simultaneously for a median of five courses. The median follow-up period was 32 months (IQR: 28 months). Grade 3 acute toxicity was observed at the gastrointestinal level in seven patients (16%). Late grade 3/4 toxicity was observed in 14 patients (33%). Seven patients (16%) persisted with the disease and five died. The local relapse rate was 9%. Eleven patients underwent a hysterectomy after treatment. The disease-free interval was 24.2 months. The 2-year global survival rate was 82.9%.

**Conclusion:**

Concurrent chemo-radiotherapy appears to be an effective regimen, with acceptable toxicity, for patients with locally advanced cervical cancer.

## Introduction

In 2012, 528,000 new cases of women with cervical cancer were diagnosed worldwide [1]. In Argentina, 4,956 women are diagnosed each year with the same disease and 2,127 die each year. Cervical cancer is the third most frequent cancer in Argentina among women (after breast and colorectal cancer) and the second most frequent among women aged 14–44 years old [2].

Cervical cancer is staged by clinical gynaecological examination. In 2009, the most up-to-date version of cervical cancer staging was published by the Committee of the International Federation of Gynecology and Obstetrics (FIGO) [3]. Since 2009, FIGO has recommended, whenever possible, an assessment with magnetic nuclear resonance since it is the best diagnostic method for locoregional staging of this cancer. This was confirmed in the recent FIGO staging published in 2018, where the imaging and pathological findings are incorporated into the staging [4].

Stage IB2 is considered to be locally advanced cervical cancer [5]. Local, regional and distant relapses are more likely in this group of patients.

In the treatment of locally advanced cervical cancer, five randomised Phase III clinical studies [6–10] and two meta-analyses [11, 12] showed significant improvement in overall survival and progression-free survival with radio-chemo-concurrence with platinum, with respect to exclusive radiotherapy, or with hydroxyurea. Because of these studies, in 1999, the National Cancer Institute (NCI) issued the recommendation for use, thus making radio-chemo-concurrence the standard treatment for cervical cancer in locally advanced stages [13]. In spite of the development and improvement in radiotherapy techniques, it is not possible to reach the dose of radiation required by the pathology without the use of brachytherapy. Although the different forms of delivery produce the same global survival rates, high-rate brachytherapy incorporated in the last decades improves the use option, facilitating the practice for the operator and improving tolerance for patients and shortening treatment time [14]. Radiotherapy is curative for locally advanced cervical cancer for approximately 65%–75% of patients in Stage IIB, for 30%–50% of patients in Stage IIIB and for 10%–15% of patients in Stage IVA. However, radiation treatment for gynaecological cancers brings to light questions about the quality of life for patients, due to its toxicity.

There is very little communication about this type of treatment in South America [15], the majority of the literature coming from other regions, [16–22] and which examine the benefits and morbidity of the treatment. Therefore, this research would provide greater knowledge regarding the treatment of cervical cancer in our region and encourage the prospective registration and dissemination of its results in Latin America. In this paper, we aim to describe the experience of the Hospital Italiano de Buenos Aires in the treatment of concurrent chemo-radiation and high-rate brachytherapy in locally advanced stages of cervical cancer through the analysis of a retrospective cohort from 2009 to 2015.

### Objectives and methods

A descriptive and observational study was carried out in the Gynecology and Radiation Oncology Department (Mevaterapia) of the Hospital Italiano de Buenos Aires.

The study population consisted of patients in the registries during the period 2009–2015 with a diagnosis of cervical cancer of epithelial origin in locally advanced stages who had received external pelvic radiotherapy with three-dimensional shaped technique (3D) plus high-rate brachytherapy and concurrence with cisplatin as primary treatment. Patients with previous pelvic radiotherapy, presence of another type of concomitant cancer, history of partial or total hysterectomy, patients who had begun their treatment in another healthcare centre and patients with a histological diagnosis of neuroendocrine carcinoma were excluded.

### Field work

The clinical histories corresponding to the selected patients were reviewed and the information was collected in a database constructed for that purpose. The following variables were evaluated for each patient: age, performance status, body mass index, previous surgeries, last pretreatment haemoglobin value, pretreatment creatinine clearance, histological tumour type, tumour size, FIGO stage, tumour characteristics in the MRI, lymph node staging by images or surgery, characteristics of the radiant treatment, characteristics of concurrent chemotherapy, variables in terms of oncological follow-up, toxicities, persistence, recurrence (with histological confirmation) and death.

We reviewed the images of the MRI performed on the patients in the context of pretreatment staging to evaluate prognostic factors, such as tumour size, involvement and location of lymph nodes, involvement of parametria, involvement of septums and invasion of other adjacent organs. The following were considered to be (1) signs of lymph node involvement: diameter greater than 1 cm on the minor axis, changes in lymph node morphology, altered signal at T2 or heterogeneous image and restricted diffusion, (2) signs of parametrial involvement: interruption in the paracervical fibrous ring or parametrium-tumour irregularity and (3) signs of septum involvement: anterior or posterior fatty infiltration.

Although there are different criteria for classifying acute [23] or chronic [24] toxicity, in this study, we defined the limit as 90 days from the beginning of treatment.

Major toxicity was reported as Grades 3, 4 and 5. Acceptable toxicity was classified as Grades 1 and 2.

Acute haematological toxicities were classified according to the NCI Common Terminology Criteria for Adverse Events, version 3.0 [25] Score, and non-haematological acute toxicities and chronic toxicities were classified using the Toxicity Criteria of the Radiation Therapy Oncology Group and the European Organization for Research and Treatment of Cancer (EORTC) [26] scores. However, this last scale may be considered to be inadequate to classify some of the more common complications of radiation treatment for cervical cancer; therefore, more variables in this category were identified using the description published in Eifel’s study [27].

Oncological monitoring data were obtained from medical visits stored in the hospital’s electronic clinical history or from telephone calls that the investigators made to patients whose last control was not carried out at the institution.

### Standard evaluation and treatment

All patients were regularly examined by gynecologists, oncologists and radiant oncologists. Pretreatment evaluation consisted of taking the clinical history, a physical evaluation and pelvic examination, a biopsy of the uterine cervix and an MRI of the abdomen and pelvis.

Staging of the lumbar aortic area to define the radiation treatment was performed in two ways: through positron emission tomography (PET) CT or through lumbar aortic laparoscopic staging surgery. The latter began at our institution under a research protocol starting in 2012.

External radiation was administered using 3D. A pelvic scan was performed with oral contrast in the institutional scanner (Philips Brilliance CT 16-slice). The images were imported into the planning system (Blue Frame Shell, Version 14.05.00). Planning was organised using anterior, posterior and both lateral fields known as the four-field box technique in the pelvic area and with anterior, posterior and lateral fields in the lumbar aortic area. Photon energy of 6, 10 and 15 MV were used. Patients received a dose of 50–50.4 Gy in 25–28 fractions in the pelvic area and 45 Gy in 25 fractions in the lumbar aortic region. Some patients received a parametrial boost of 5.4 Gy in three additional fractions. Regarding brachytherapy—until July 2015, iridium 192 was used as a radioactive source using 2D, following the international guidelines (International Commission on Radiation Units and Measurements (ICRU) 38) [28]. Then, it was replaced by cobalt 60 using 3D and applicators compatible with computed axial tomography. The volumes to be treated were delineated according to the consensus of the Groupe Européen de Curietherapie and the European Society for Radiotherapy and Oncology [29]. In both 2D and 3D, a 6-Gy design per fraction was used for a total dose of 24 Gy. Two fractions per week were given, making a total of four fractions.

Patients received indications for concurrent chemotherapy with cisplatin at a dose of 40 mg/m2 (maximum dose of 70 mg) once a week for five cycles during the period of radiation therapy. Our scheme is coincident with what is described in the current literature [7, 9, 30, 31]. The causes for suspension of chemotherapy were as follows: (1) fever over 38°C, (2) neutropenia under 1,500/mm^3^, (3) thrombocytopenia under 75,000/mm^3^, (4) vomiting, (5) diarrhoea, (6) creatinine clearance dropping to under 50 mL/minute and (7) performance status 2 or more. Chemotherapy was resumed when toxicity became Grade 1. In premedication, patients received hydration with saline solution, mannitol solution, ranitidine 50 mg IV, dexamethasone 8 mg IV and ondansetron 8 mg IV.

Laboratory tests with blood count, liver function tests, electrolytes and creatinine clearance were requested at the initial evaluation and repeated at 3 weeks. A weekly clinical check-up was performed during treatment. Then, patients were monitored every 3 months for the first 2 years and every 6 months until the end of the 5 years. After treatment, a control study with images was requested. The negative, positive and doubtful findings in the images were described. We consider doubtful findings to be inflammatory changes that could not be classified and that required a new control after a prudent period of time (more than 3 months).

### Statistical analysis

In the descriptive statistical analysis, the continuous variables were expressed as median and interquartile range (IQR), and the categorical variables were summarised as absolute and relative frequencies.

In the follow-up analysis, the follow-up time was calculated as the difference between the date of diagnosis and the date of the final control or death. Three patient conditions were analysed: death by illness, persistence (patients who remained actively ill with cancer in spite of treatment) and relapse (reappearance of the illness in patients who responded to treatment).

For the death event, the fatality rate was estimated as the number of deceased patients per 100 persons with cancer and overall survival at 2 years as a percentage of living patients per 100 patients with cervical cancer, with their respective confidence intervals of 95%. Patients followed for less than 2 years were excluded from the analysis (*n* = 35).

For relapse, the rate was estimated to be the number of relapsed cases per 100 person-years, with a confidence interval of 95%, and the period free of illness as the time from diagnosis of the illness until the date of the first relapse, last medical control or death, expressed as median in months and interquartile range. Patients with less than 2 years of follow-up were excluded from the calculation (*n* = 32).

The percentage of tumour persistence and death was calculated for the total cohort.

Statistical analysis was performed using the STATA 13 program.

The clinical study was approved by the Ethics Committee of the Hospital Italiano de Buenos Aires, Argentina.

## Results

A total of 43 patients were included. The patients’ characteristics are listed in Table 1. Six patients (14%) began treatment with a haemoglobin level of less than 10 g/dL. Of the total, five patients (11%) required a blood transfusion prior to treatment. Seven (16%) patients began with creatinine clearance under 70 mL/minute.

Table 2 shows the tumour details. Thirty-eight patients (88.4%) had a cervical tumour size of ≥ 4 cm. Twelve patients (28%) had a tumour size of ≥ 6 cm. Images showed lymph node involvement in the pelvic area in 19 patients and in the lumbar aortic area in only one patient. Lymph node involvement was shown in both areas in only two patients. Parametrial involvement was detected by MRI in 24 patients in stages II and III and five patients in stages IB and IIA; in these cases, the parametrial involvement was not detected in the physical examination. Septum involvement was observed in 12 patients (28%) by MRI. Lumbar aortic lymph node staging was accomplished through laparoscopic surgery in 23 patients (23/43), nine of which showed involvement (9/23), and through PET in 20 patients (20/43), of which six were positive (6/20). In all, only two patients did not have lumbar aortic staging.

Table 3 shows the radiation treatment details. Regarding the duration of radiation treatment (external and brachytherapy), the median was 58 days (interquartile range: 20 days). A total of 29 patients (67.4%) completed concurrent radiochemotherapy in ≤ 63 days. For most patients (95%), treatment was sequential. One patient required hemostatic flash as part of the treatment due to anaemia at the time of presentation, and three patients had a boost in parametrium. Seventeen patients had extended-field radiation: 15 for lymph node involvement (surgery and PET) and two patients for massive pelvic lymph node involvement. The median duration of extended-field radiation was 41 days (interquartile range: 6 days). Regarding the chemotherapy, the median for cycles completed was five cycles. Only seven (16%) patients had to suspend chemotherapy. The principal causes were diarrhoea, decreased creatinine clearance and vomiting.

Table 4 lists the frequency of acute and chronic toxicities selected for this study, according to the highest grade reached. Although most patients experienced acute gastrointestinal or urinary toxicity, only 16% experienced severe toxicity (Grade 3) in the digestive tract. Only two patients experienced Grade 4 acute toxicity: one patient with debilitating sensitive neuropathy and one patient with septic shock secondary to pyometra. In addition to the toxicities reported in the table, 12 patients (28%) experienced some acute toxicity: pelvic pain (*n* = 1), vaginal disorders (*n* = 3), hot flashes (*n* = 3), bone fractures (*n* = 1) and hydroelectrolyte changes (*n* = 4). Of the total, only five patients required hospitalisation.

When we group toxicities by patient, we see that 14 patients (33%) experienced some Grade 3/4 chronic toxicity. Of these, most had symptoms in the rectosigmoid area with pain and urgency. Intestinal and urinary toxicities appeared with a median time from the end of treatment of 10.8 (IQR: 4.9) and 10.8 (IQR: 14.7) months, respectively. Of the total, only 11 patients required hospitalisation. There were other chronic toxicities not reported in Table 4: bone fracture (*n* = 1), menopause (*n* = 8), thrombosis (*n* = 2), bone marrow depression, pyometra/hematometrea, pelvic pain and edema.

Four patients experienced fistulas: two rectovaginal and two vesicovaginorectal. Median time to onset of the fistula was 9.5 months (IQR: 1.6). In two patients, the fistula was due to tumour persistence.

The median time to the first control with images was 2.7 months. Forty-one patients had MRIs (95%) and negative findings were seen in 51%, positive findings in 23% and doubtful findings in 26%.

After treatment, ten patients (23%) received a cervical biopsy for suspected persistence of the disease (clinical or MRI), of which four were positive for the disease. Eleven patients (26%) had a rescue hysterectomy with the following indications: pyometra, persistent metrorrhagia, probable or clearly clinically persistence, persistence confirmed by pathology. Of the hysterectomies performed, three were positive for cancer (3/11).

The oncological results are shown in Table 5. Of the total, 29 patients (67%) had a follow-up time of > 2 years, with a minimum of 6 months (due to death from the disease) and a maximum of 78 months.

## Discussion

In this paper, we report the experience of 43 patients with pelvic 3D-CRT and high-rate brachytherapy with concurrent chemotherapy in advanced cervical cancer. At 2 years, the global survival rate was 82.9%, with a tumour persistence rate of 16% and local recurrence of 9%. With a median follow-up time of 32 months, grade 3 acute gastrointestinal toxicity was observed in 16% and grade 3/4 chronic toxicity in 33%.

Table 6 is a comparison with other studies available from the current literature. There were wide differences in treatment, recording, definition and evaluation of toxicities which hinder the comparative analysis.

It is well known that the total time of treatment is one of the most important prognostic factors in patients with cervical cancer treated with radiotherapy. In this sense, two studies have shown a significant decrease in pelvic control and the overall survival rate when the treatment exceeds 6 weeks [21, 32]. In this study, although all the patients completed the proposed radiant treatment, 14 of them did it in a longer time than recommended. This was probably due to the availability of brachytherapy equipment, to delays due to non-working days and to special situations on the part of patients, such as non-medical situations and patients residing long distances from the radiation centre.

On the other hand, in July 2015, the radiotherapy department acquired a brachytherapy device with 3D planner, which improved the combination of the two radiant treatments, improving therapeutic times. This practice provided a reduction in the toxicity observed in patients treated after this date. Modulated Intensity Radiotherapy and Modulated Intensity Volumetric Arcotherapy are currently being incorporated into the treatment of cervical cancer. It will be interesting to know in the future how this technology influences the survival and toxicity of the patients treated.

Regarding acute toxicity, Toita observed that grade 3/4 leukopenia was the most frequent adverse effect and found a 25% incidence in grade 3/4 toxicity in platelets. [21] Potter found 4% grade 3 anaemia, 23% grade 3/4 leukopenia and 10% grade 3 thrombocytopenia [33]. Al Asiri reported 5% grade 3/4 leukopenia [22]. Unlike previously published results, our work showed less acute haematological toxicity, the majority being grade 1/2 and only 9% grade 3 leukopenia. Toita reported 28% grade 3/4 nausea and vomiting and 15% grade 3/4 diarrhoea [21]. Al Asiri observed 5% grade 3/4 nausea and vomiting [22]. Our work yields almost zero toxicity at the level of nausea and vomiting and 16% grade 3/4 diarrhoea; this coincides with what has been reported.

As for chronic toxicity, gastrointestinal symptoms are the most commonly observed adverse effects after pelvic radiotherapy. More than 50% of patients experienced some form of this. The average time of presentation is 8–10 months after treatment [24]. Ogino reported a late rectal complication rate for grades 1, 2, 3 and 4 of 5.6%, 31.7%, 6.8% and 8.1%, respectively [34]. Our study yields similar results: 4.7%, 25.6%, 11.6% and 9.3%, respectively. Likewise, Souhami reported severe toxicity after treatment: rectal ulcer (*n* = 10), obstruction of the small intestine (*n* = 2) and recto-vaginal fistula (*n* = 2) [17]. Other authors reported less chronic intestinal toxicity. Toita observed eight patients (20%) with gastrointestinal complications (all grades) [21]. Potter did not report any late toxicity at the gastrointestinal level grade 3/4 [33]. On the other hand, Nakano conducted a long-term follow-up (22 years) and reported major colorectal complications at 5, 10 and 20 years at a rate of 3.8%, 4.4% and 5.3%, respectively [35]. These results warn of the possibility of long-term toxicity.

The urological effects are mild; the most common are the narrowing of the urethra and actinic cystitis [24]. However, they increase in frequency over time. Nakano reported major urological complications at 5, 10 and 20 years at rates of 0.8%, 0.9% and 1.3%, respectively [35]. In our study, chronic toxicity at the urinary level was 16% and only two patients had severe toxicity (grade 4), in the same proportion as reported by Potter [33].

The impact of the radiation therapy on bones is recognised and can manifest itself in different ways. The most common event is a pelvic fracture, which may be asymptomatic and can be diagnosed with images. The mean time to the event is 6–20 months after radiation ends [24]. We found two asymptomatic cases in our series, the first was an L4 vertebral fracture that required vertebroplasty. The second was osteonecrosis of the symphysis pubis and a sacral fracture that improved with medical treatment.

Pelvic radiotherapy is especially toxic for the ovaries in premenopausal patients and can lead them to experience early menopause. It has been reported that vasomotor and genitourinary symptoms (atrophy and dysfunction) of menopause affect 80% of patients and worsen their quality of life [24]. Eleven patients in our study reported hot flashes and vaginal dryness and nine presented dyspareunia or vaginal shortening. These conditions are related to treatment and to the resulting hormonal disorder. We believe that these values are underrepresented because this toxicity is very difficult to evaluate and requires systematization, with a direct interrogation to achieve adequate understanding. This is not yet incorporated into our usual practice. Due to the importance it has for patient quality of life, we consider the search of symptoms and the interrogation of the patient very important in the follow-up and treatment.

In the context of toxicities, it is notable that 39% of patients had extended-field radiation and 26% has post-radiation surgery. Both factors consistently influence toxicity rates, causing them to rise.

The frequency of delayed toxicity observed in our study was higher than in other studies. This finding could result from careful, systematic follow-up of patients after radiotherapy to detect possible sequelae, including uncommon adverse effects.

Follow-up time was short, but this study found a high overall early survival rate and a low local relapse rate. This finding is likely due to several factors: rigorous patient selection in an interdisciplinary context, the firm belief of the medical team in the treatment, following guidelines that limited treatment volume, and careful planning of clinical monitoring between treatments. These factors all contribute to treatment compliance and good adherence by patients.

For study treatments, dose prescription and planning were adjusted based on the aforementioned principles. Last, it is notable that a considerable percentage of patients were in stages 1B or 2 (*N* = 12). This fact contributed to better oncological results and a lower possibility of treatment-associated adverse effects.

Some surgeons complete hysterectomies after radiotherapy in selected patients (large initial tumour size, residual post-treatment disease). Results of the GYNECO 02 study suggest that performing a post-radiation hysterectomy has no therapeutic impact on patients with a complete clinical and radiological response [36]. In our study, 11 patients had surgery after treatment, which involves greater surgery-associated morbidity for patients. Reducing the number of post-radiation surgeries is thus desirable.

Examining histological tumour type, our study found that most were epidermoid (*N* = 37), and very few were of other histological types. It was therefore not possible to know and document if there are differences in radiotherapy treatment results.

This study has several limitations. It describes results in a single, private centre with a small number of patients, making it difficult to extrapolate those results to other centres. However, it would be interesting to conduct a national, multi-centre study. Conversely, complications and grade 1 and 2 toxicities may have been underreported in this study. Underreporting may have resulted from problems following and recognising related post-treatment symptoms. Moreover, delayed toxicities may not be represented as some patients did not survive long enough to experience delayed complications of radiation. Another limitation of this study is that it was retrospective and patient case histories may have contained incomplete records. Finally, the follow-up time in the study was short for evaluating oncological results and delayed toxicities.

However, we can also highlight the strengths of this study. They include a homogeneous population with a reasonable number of patients from a single institution in Argentina. That facility used modern radiation technology and collected information by telephone, not losing any patients to follow-up.

## Conclusion

This study provides us with us an initial updated approach of primary radiotherapy in in cervical cancer patients with locally advanced stages at Hospital Italiano de Buenos Aires.

The delayed adverse effects most commonly observed in our population were gastrointestinal symptoms. The rate of acute toxicity was lower than expected, while the rate of chronic toxicity was higher due to a correct and adequate follow-up of our patients and knowledge on the part of the patients, with the purpose of an accurate diagnosis and precise handling of the sequela of radiant treatment. It would be interesting to compare these findings with the results of other studies in Latin America. This study contributes to our understanding of the current situation of patients and available treatments in the region.

With our follow up period, the overall early survival rate is higher than what is reported in other internationally published studies.

In conclusion, we report favourable results regarding radiochemotherapy concurrent with high rate brachytherapy in cervical cancer in locally advanced stages. This concurrent treatment showed good local control, was well tolerated, and had an acceptable percentage of complications.

We believe that we will benefit from new advances and treat cancer more effectively using multi-modal treatments, molecular therapies and fewer radical surgeries, with lower morbidity.

## Conflict of interest

The authors do not have any conflicts to disclose.

## Funding statement

No financial funding was provided to support this project.

## Figures and Tables

**Table 1.** Baseline characteristics of cervical cancer patients (*n* = 43).

Details	Measurement
Patients (*n*)	43
Age (years), median (IQR)▵	45 (26)
Performance status (*n*/%)	
0	32/74.4
1	10/23.3
2	1/2.3
Body Mass Index in kg/m2, median (IQR)▵	23.8 (7.5)
Patient with history of prior surgeries (*n*/%)	
No	30/69.8
Yes	13/30.2
Haemoglobin pretreatment in g/dL, median (IQR)▵	12 (2.1)
Creatinine clearance in mL/min, median (IQR)▵	97 (47.7)

▵ Interquartile range

**Table 2.** Tumour characteristics of cervical cancer patients (*n* = 43).

Details	Measurement
Histological type (*n*/%)	
Squamous	37/86
Adenocarcinoma	4/9.3
Other	2/4.7
Tumour size in cm, median (IQR)▵	45 (20)
Tumour stage (FIGO) (*n*/%)	
IB2	12/27.9
IIA2	6/14.0
IIB	15/34.9
IIIB	10/23.3
Tumour size in images (MRI) in cm, median (IQR)▵	52 (17)
Lymph node involvement in images (MRI) (*n*/%)	
Negative	21/48.8
Positive	22/51.2
Parametrial infiltration in images (MRI) (*n*/%)	
Negative	14/32.6
Positive	29/67.4
Pretreatment lumbar aortic staging (surgery) (*n*/%)	
No	20/46.5
Yes	23/53.5
Lumbar aortic lymph node involvement (PET/surgery) (*n*/%)	15/36

▵ Interquartile range

**Table 3.** Characteristics of concurrent radiochemotherapy in cervical cancer patients (*n* = 43).

Details	Measurements
Radiation treatment (*n*/%)	
External RT and BT	26/60.5
External RT + BT + lumbar aortic field (group 2)	17/39.5
Radiotherapy dose (cGy) (*n*/%)	
External	
46	1/2.3
48.6	2/4.7
50	20/46.5
50.4	17/39.5
55.8	3/7.0
External radiotherapy dose fraction (cGy) (*n*/%)	
1.8	22/51.2
2	21/48.8
Radiotherapy energy (*n*/%)	
External	
F6Mv	23/53.5
F10Mv	10/23.3
F15Mv	10/23.3
Lumbar aortic field	
F6Mv	8/47.1
F10Mv	5/29.4
F15Mv	4/23.5
Brachytherapy source (*n*/%)	
Ir192	33/76.7
Co60	10/23.3
Total treatment time (days)	
External RT, median (IQR)▵	40 (7)
External RT + BT, median (IQR)▵	58 (20)
External RT + BT + lumbar aortic field, median (IQR)▵	84 (19)
Patient with total treatment time (<63 days) (*n*/%)	29/67.4
Patients receiving ≥ four cycles of chemotherapy (*n*/%)	39/90.6

RT: radiotherapy, BT: brachytherapy,

▵ Interquartile range

**Table 4.** Acute and chronic toxicities profile in cervical cancer patients (*n* = 43).

Acute adverse effect	Grades (*n*)				
	0	1	2	3	4
Lower gastrointestinal	1	5	30	7	—
Urinary	1	17	25	—	—
Asthenia	1	21	20	1	—
Dermatological	18	23	2	—	—
Vomiting	39	3	1	—	—
Anaemia (haemoglobin)	13	19	11	—	—
Leukopenia	14	19	6	4	—
Neutropenia	33	4	6	—	—
Thrombocytopenia	39	4	—	—	—
Infectious	39	—	3	—	1
Motor neuropathy	42	—	—	1	—
Sensory neuropathy	39	—	2	1	1
Abnormal transaminases	40	1	2	—	—
Abnormal bilirubin	43	—	—	—	—
Hyponatremia	36	4	—	3	—
Abnormal creatinine clearance	30	6	7	—	—
**Chronic adverse effect**	**Grades (*n*)**				
	**0**	**1**	**2**	**3**	**4**
Extremities/Neuromuscular	39	1	2	—	1
Hematochezia	21	5	12	4	1
Rectosigmoid abnormalities	21	2	11	5	4
Diarrhoea	29	4	7	3	—
Vaginal abnormalities	28	6	9	—	—
Urinary abnormalities	36	2	3	—	2

**Table 5.** Results of oncological follow-up of cervical cancer patients (*n* = 43).

Follow-up	Measurement
Follow-up time in months, median (IQR)▵	32 (28)
Fatality rate (person-years)	5/100
Global survival at 2 years (%)	82.9
Relapse rate (person-years)	5.7/100
Recurrence (*n*)	6/35
Local recurrence rate (%)	9
Time to relapse in months, median (IQR)▵	20.9 (9.26)
Disease-free interval in months, median (IQR)▵	24,2(24,2)
Persistence (*n*/ total)	7/43
Deaths (*n*/ total)	6/43

▵ Interquartile range

**Table 6.** Comparison of studies available in current literature.

Study, year		Souhami et al [17], 1993	Toita et al [21], 2005	Pötter et al [33], 2006	Atahan et al [16], 2007	Parker et al [18], 2009	Kato et al [20], 2010	Al Asiri et al [22], 2013	Giavedoni, 2016
**QT/RT concurrency (n)**		50	40	48	89/183	92	120	74	43
**Country**		Canada	Japan	Austria	Turkey	UK	South-east Asia	Saudi Arabia	Argentina
**FIGO stage (I, II, III, IV) (%)**		0, 40, 54, 6	2, 33, 65, 0	4, 50, 42, 4	10, 64, 26, 0	8, 63, 26, 3	0, 0, 50, 50	0, 66, 26, 8	28, 49, 23, 0
**Radiant treatment used**	**Technique**	Conventional (2D)	Conventional (2D) with 18 MV	CT based 3D conformal	Conventional (2D)	Conventional (2D)	Conventional (2D) ALE/60 CO	CT based 3D conformal	CT based 3D conformal
	**EBRT (DT) Gy**	46	50	50	50	45	50	45–50.4	50
	**Fields**	four-field box	APPA	four-field box	four-field box o APPA	four-field box	four-field box or APPA	APPA	four- field box
	**Boost**	—	yes	—	—	—	yes	—	yes
	**Dose Boost (Gy)**	—	6–10	—	—	—	10-15	—	5.4
	**Brachytherapy (BT)**	HDR-BT 30 Gy (3 × 10 Gy)	HDR-BT 18 Gy (3 × 6 Gy)	MRI- based 3D HDR-BT (5–6 o 7 ×7 Gy)	HDR-BT 28 Gy (4 × 7 Gy)	HDR-BT 24 Gy (4 × 6 Gy)	LDR-BT or HDR-BT	HDR-BT 28 Gy (4–6 ×7 Gy)	HDR-BT/ TAC- based 3D 24 Gy (4 × 6 Gy)
**Follow-up**	**Survival rate Early global (months)**	65% to 44	79% to 36	61% to 36	—	72% to 24	79.6% to 24	—	82.9% to 24
	**Rate of SG to 5 years (%)**	—	—	—	55	55	—	64.5	—
	**Median of Follow-up (months)**	27	37	33	45	26	27.3	60	32
	**Rate of Local relapse (%)**	7 (16%)	3 (7%)	10 (21%)	48 (26%)	—	14 (12%)	8 (11%)	3 (9%)
**Toxicity**	**Classification of toxicity (acute// chronic)**	RTOG// Pilepich%	NCI-CTC version 2.0// RTOG/EORTG	NCI// LENT SOMA score	RTOG/ EORTG// RTOG/ EORTG	// NCI-CTC version 3.0	NCI-CTC version 3.0// RTOG/ EORTG	NCI-CTC version 2.0// RTOG/ EORTG	NCI-CTC version 3.0 y RTOG/ EORTG// RTOG/ EORTG
	**Late toxicity G3 or G4 (%)**	13/50 (26)	1/40 (2.5)	2 (4)	8/183 (8)	4/92 (4)	3/120 (4)	3 (4)	14/43 (32.5)
	**Areas evaluated - Chronic toxicity**	bladder, bowel	bladder, bowel	vagina, bladder, bowel	bladder, bowel	vagina, bladder, bowel, neuromusc/ ext	bladder, bowel	urinary tract, bowel, neuropathy, hearing loss	vagina, bladder, bowel, neuromusc/ ext

QT/RT: chemoradioconcurrence, FIGO: International Federation of Gynecologists and Obstetrics, CT: computed tomography, 3D: three dimensional, ALE: linear accelerator, 60CO: cobalt 60, Gy: grey, APPA: anterior-posterior, HDR- BT: high rate brachytherapy, LDR- BT: low rate brachytherapy, MRI: magnetic resonance imaging, SG: global survival, RTOG: Radiation Therapy Oncology Group, NCI-CTC: Common Toxicity Criteria of the National Cancer Institute, RTOG/EORTG: European Organization for Research and Treatment of Cancer, LENT SOMA: Late Effects Normal Tissue Task Force- Subjective, Objective, Management, Analytic scales, Neuromuscular/ext: neuromuscular/extremities % Pilepich, M. International Journal of Radiation Oncology, Biology, Physics. 1987; 13 (3):351–357, EBRT: External Beam Radiation Therapy, DT: Total dose, TAC/TC/CT: computed axial tomography
